# *Cis*- and *Trans*-Palmitoleic Acid Isomers Regulate Cholesterol Metabolism in Different Ways

**DOI:** 10.3389/fphar.2020.602115

**Published:** 2020-12-08

**Authors:** Wen-wen Huang, Bi-hong Hong, Kai-kai Bai, Ran Tan, Ting Yang, Ji-peng Sun, Rui-zao Yi, Hao Wu

**Affiliations:** ^1^ Third Institute of Oceanography, Ministry of Natural Resources, Xiamen, China; ^2^ Department of Pharmacology, School of Pharmacy, Nanjing University of Chinese Medicine, Nanjing, China; ^3^ Fujian Provincial Key Laboratory on Hematology, Department of Hematology, Fujian Medical University Union Hospital, Fujian Institute of Hematology, Fuzhou, China; ^4^ Zhejiang Marine Development Research Institute, Zhoushan, China

**Keywords:** *cis*-palmitoleic acid, *trans*-palmitoleic acid, cholesterol, absorption, hypercholesterolemia, cardiovascular disease

## Abstract

Hypercholesterolemia is a preventable risk factor for atherosclerosis and cardiovascular disease. However, the mechanisms whereby *cis*-palmitoleic acid (*c*POA) and *trans*-palmitoleic acid (*t*POA) promote cholesterol homeostasis and ameliorate hypercholesterolemia remain elusive. To investigate the effects of *c*POA and *t*POA on cholesterol metabolism and its mechanisms, we induced hypercholesterolemia in mice using a high-fat diet and then intragastrically administered *c*POA or *t*POA once daily for 4 weeks. *t*POA administration reduced serum cholesterol, low-density lipoprotein, high-density lipoprotein, and hepatic free cholesterol and total bile acids (TBAs). Conversely, *c*POA had no effect on these parameters except for TBAs. Histological examination of the liver, however, revealed that *c*POA ameliorated hepatic steatosis more effectively than *t*POA. *t*POA significantly reduced the expression of 3-hydroxy-3-methyl glutaryl coenzyme reductase (HMGCR), LXRα, and intestinal Niemann-Pick C1-Like 1 (NPC1L1) and increased cholesterol 7-alpha hydroxylase (CYP7A1) in the liver, whereas *c*POA reduced the expression of HMGCR and CYP7A1 in the liver and had no effect on intestinal NPC1L1. In summary, our results suggest that *c*POA and *t*POA reduce cholesterol synthesis by decreasing HMGCR levels. Furthermore, *t*POA, but not *c*POA, inhibited intestinal cholesterol absorption by downregulating NPC1L1. Both high-dose *t*POA and *c*POA may promote the conversion of cholesterol into bile acids by upregulating CYP7A1. *t*POA and *c*POA prevent hypercholesterolemia via distinct mechanisms.

## Introduction

Cholesterol is one of the main mediators of cardiovascular pathobiology, and hypercholesterolemia is a well-established cause of cardiovascular morbidity and mortality worldwide (Gielen and Landmesser, 2014; Catapano et al., 2016). Hypercholesterolemia has a mean mortality rate of 21.1%, and about 17.8 million people died from cardiovascular diseases worldwide in 2017 (GBD 2017 Causes of Death Collaborators, 2018). Hypercholesterolemia increases the risk of coronary heart disease and atherosclerosis (Yu et al., 2019) and frequently coexists with hypertension, obesity, and hyperglycemia. There are several other frequent comorbidities linked with hypercholesterolemia, including fatty liver disease, gallstones, diabetes, Alzheimer’s disease, and some cancers. Cholesterol is absorbed in the intestine via its biosynthesis in the liver and from dietary intake. Therefore, the inhibition of cholesterol biosynthesis, its intestinal absorption and the promotion of cholesterol excretion represent therapeutic approaches to counteract hypercholesterolemia.

It is known that 70–80% of the body’s cholesterol is synthesized by the liver and 10% by the small intestine and that 3-hydroxy-3-methyl glutaryl coenzyme reductase (HMGCR) is a rate-limiting enzyme in cholesterol synthesis (Altmann et al., 2004). Statins are significant inhibitors of HMG-CoA reductase. Most lipids from the diet and other nutrient molecules are absorbed via specific transporters on the brush border membrane of small intestinal enterocytes. Niemann-Pick C1-Like 1 (NPC1L1) is a transporter for the absorption of cholesterol (Altmann et al., 2004; Xie et al., 2012). Cholesterol-binding activity facilitates the movement of cholesterol between the endocytic recycling compartment and the plasma membrane so that it can be transported into cells by clathrin-mediated endocytosis (Zhang et al., 2011; Kamishikiryo et al., 2017) and cholesterol absorption is lower in NPC1L1-knockout mice (Temel et al., 2007; Liqing, 2008). Total bile acid (TBA) was derived from endogenous synthesis and intestinal absorption. Bile acid is synthesized in the liver, nearly 50% of an adult body’s synthetic cholesterol in a day is excreted following catalytic conversion into bile acids (BAs) (Fan and Guo, 2011). Cholesterol 7-alpha hydroxylase (CYP7A1) is a rate-limiting enzyme in the synthesis of BAs from cholesterol in the liver. It therefore plays an important role in the maintenance of cholesterol homeostasis and BA synthesis.

LXRα, a member of the nuclear receptor superfamily, acts as a cholesterol metabolite receptor in the liver and kidneys. It regulates the absorption, transport, and transformation of cholesterol by regulating its target genes. CYP7A1 is a target gene of LXRα in rodents (Xie et al., 2012), and a recent study showed that activated LXRα regulates NPC1L1 expression to promote cholesterol metabolism (Li et al., 2018).

Palmitoleic acid (POA) is a 16-carbon monounsaturated fatty acid with a double bond between its seventh and eighth carbon atoms (16:1, n-7, molecular weight 254.42). Most studies on POA have focused on *cis*-POA (*c*POA) or natural mixtures containing *c*POA, rather than *trans*-POA (*t*POA). *c*POA is thought to mainly exist in the liver and adipose tissue, from where it can act as a lipid signaling substance to regulate lipid metabolism by regulating plasma low-density lipoprotein (LDL-c) and total cholesterol (TC) concentrations and by regulating triglyceride (TG) biosynthesis by affecting the expression of fatty acid-binding protein (Sansone et al., 2013; Rafacho and Nunes, 2017). *c*POA intake is inversely proportional to gastric emptying time and affects gastrointestinal transportation and appetite. Consumption of *c*POA or materials containing it promotes the secretion of satiety hormones in mice, reduces the accumulation of cholesterol and lipoprotein in the aorta and lipids in the plasma, which prevents atherosclerosis, fatty liver, and metabolic disorders caused by a high-fat, high-carbohydrate diet (Yang et al., 2013; Souza et al., 2014; Rafacho and Nunes, 2017).

The level of *t*POA consumption correlated with plasma LDL-c concentrations in a study of atherosclerosis conducted in participants from multiple ethnicities and oral *t*POA reduced the risk of diabetes and atherosclerosis in individuals consuming high-fat diets (HFDs) (Mozaffarian et al., 2010a; Mozaffarian et al., 2010b). *t*POA has also been shown to inhibit the appetite, improve insulin sensitivity, and reduce fat accumulation in the abdominal cavity of obese mice without any adverse effects (Wang, 2008). Additionally, the plasma concentration of phospholipid containing *t*POA was found to be inversely proportional to hepatic fat content (Li et al., 2019). Finally, Bolsoni *et al.* (Bolsoni-Lopes et al., 2013) found that POA regulates the biosynthesis of triglycerides in adipocytes, increases lipolysis, inhibits hepatic steatosis, and accelerates glucose metabolism by regulating the activity of peroxisome proliferator-activated receptor (PPAR)α and thereby that of the enzymes encoded by its target genes. Nevertheless, it is unclear whether *c*POA or *t*POA can ameliorate hypercholesterolemia by affecting the activity of LXRα and the downstream expression of NPC1L1 and CYP7A1.

Therefore, the purpose of the present study was to compare the effects of *c*POA and *t*POA on cholesterol biosynthesis and its absorption and excretion and to determine the mechanisms whereby these agents might ameliorate hypercholesterolemia. We provide experimental data to guide the rational use of POA or fish oils containing *c*POA and *t*POA.

## Material and Methods

### Experimental Animals

We studied db/db mice, which are leptin receptor (Lepr)-deficient and are characterized by disordered lipid metabolism, obesity, hypertension, and diabetes. They had high serum cholesterol concentrations (10–20 mmol/L), but wide individual variation; therefore, they were provided with a HFD to further raise their circulating cholesterol concentrations. In this way, we generated a model of clinical diabetes with hyperlipidemia. In female db/db mice, the masses of the uterus and ovaries are low, as is the secretion of female sex hormones, such that homozygous mice are sterile. Therefore, the mice are maintained on a Dock7m background, which is characterized by a normal serum cholesterol concentration; therefore, db/m mice are commonly used as controls.

Sixty db/db mice (specific pathogen-free, males, 15 weeks of age) and ten db/m mice were purchased from Changzhou Cavens Laboratory Animal Co. Ltd (Jiangsu, China). The mice were multiple-housed in filter-top cages at 23 ± 2°C, with a 12-h light/dark cycle. They had *ad libitum* access to food and water throughout the study period. At the end of the study, they were euthanized by cervical dislocation. All animal experiments were performed in accordance with the guidelines of Nanjing University of Chinese Medicine for the ethical care of animals and were approved by the Medical Ethics Committee of Nanjing University of Chinese Medicine. The study was conducted in accordance with the National Institutes of Health Guide for the Care and Use of Laboratory Animals (NIH Publications No. 8023, revised 1978).

### Reagents

*c*POA and *t*POA were manufactured in our laboratory (≥99%, CAS: 373-49-9, CAS: 10030-73-6). Simvastatin was purchased from Hangzhou MSD Pharmaceutical Co., Ltd., China (No. 35508). Anti-NPC1L1 antibody, anti-LXRα antibody, anti-CYP7A1 antibody, and anti-β-actin antibody were purchased from Abcam (Burlingame, CA, USA) or Cell Signaling Technology (Danvers, MA, USA). TG, TC, high-density lipoprotein-cholesterol (HDL-c), LDL-c, free cholesterol (FC) and total bile acid (TBA) measurement kits were purchased from Nanjing Jiancheng Biotechnology Co. Ltd., China.

### cis-palmitoleic acid and trans-palmitoleic acid Preparation Method

*c*POA and *t*POA were separated and puritified with column chromatography and preparative liquid chromatography from fish oil (Huang et al., 2018a), and the purity of which determined by high performance liquid chromatography (HPLC) (Huang et al., 2018b) were both more than 99%.

### Animal Groups and Diets

After 3 weeks of acclimation, the 10 db/m mice continued to consume a normal diet and the 60 db/db mice were started on a HFD to induced hypercholesterolemia. After 3 weeks on these diets, the serum cholesterol concentration of each mouse was measured and the mice were allocated to the following seven experimental groups (n = 10): control group, HFD group, *c*POA groups intragastrically administered with 150 mg/kg/mouse or 300 mg/kg/mouse of *c*POA, *t*POA groups intragastrically administered with 150 mg/kg/mouse or 300 mg/kg/mouse of *t*POA, simvastatin group intragastrically administered simvastatin at 5.2 mg/kg/mouse in saline (contains 5% ethyl alcohol), daily. The mice were treated for 4 weeks.

Our previous studies have shown that the food intake of mice administered *t*POA begins to change after 1.5 weeks and is significantly different from that of the control group after 4 weeks. Therefore, in the present study, we measured food intake during the fourth week of treatment. At this time, each mouse was individually housed, provided with the same amount of food and the mean food intake of each group was calculated. The HFD comprised 10 lard, 10 sugar, 5 protein, 0.5 cholesterol, and 74.5 base feed by mass.

### Sample Collection and Measurements

#### Serum Sample Preparation and Measurements

Before the final intragastric administration, the mice were fasted for 12 h. Then, 1 h after the treatment they were anesthetized with 40–80 mg/kg phenobarbital and the whole blood drawn from the retro-orbital sinus of each mouse was placed into a centrifuge tube without anticoagulant. The blood samples were stored at room temperature for 30–60 min and the serum was separated by centrifugation at 1,000 × *g* at 4°C for 10 min. Serum samples were stored at −80°C until the TC, TG, HDL-c, and LDL-c concentrations were measured.

#### Tissue Collection

After blood collection, the intestinal and liver tissues were quickly excised and washed with cold physiological saline. Portions of each liver were fixed in 4% paraformaldehyde solution prior to hematoxylin and eosin (HE) staining and intestinal and liver samples were snap-frozen in liquid nitrogen and stored at −80°C until their FC and TBA concentrations were measured.

#### Hematoxylin and Eosin Staining and Liver Lipid Assay

Paraffin sections of liver tissue were stained using HE. According to the product’s instructions, 50 mg of liver tissue was homogenized in 1 ml ethyl alcohol lysis buffer, the homogenate was centrifuged at 3000 × *g* for 10 min at 4°C and the supernatant was separated and used for protein quantification and FC and TBA concentration measurements.

#### RNA Isolation and Real-Time PCR

These procedures were performed as previously described (Wahlström et al., 2016). Total RNA was extracted from liver and intestinal samples using Trizol reagent (Thermo Fisher Scientific, Wilmington, USA), according to the manufacturer’s instructions. The RNA concentrations were measured using Nanodrop 2000c (Thermo Fisher Scientific, Wilmington, USA), after which the RNA was reversed transcribed using 5× Hifair Strand cDNA Synthesis SuperMix (Yeasen Biotech, China). Real-time PCR was performed using SYBR Green (Thermo Fisher Scientific, Wilmington, USA) and LC480 (Roche, Mannheim, Germany). The relative expression levels of each target gene were calculated using a standard curve and normalized to the expression of the reference genes (*Actb* and *Gapdh*). All experiments were repeated at least three times. The primers were synthesized by Generay Biotech, China. The primer sequences are shown in Table 1.

**TABLE 1:** Primers used for real-time PCR analysis.

Gene	Forward primer	Reverse primer
*β-actin*	5′–TATGCTCTCCCTCACGCCATCC–3′	5′–GTCACGCACGATTTCCCTCTCAG–3′
*GAPDH*	5′–ATCATCTCCGCCCCTTCTG–3′	5′–GTGATGGCATGGACTGTGG–3′
*LXRα*	5′–AGATCGCCTTGCTGAAGACC–3′	5′–ATGCTCTCACTCCCAGGGTT–3′
*HMGCR*	5′–CCTCCATTGAGATCCGGAGG–3′	5′–AAGTGTCACCGTTCCCACAA–3′
*NPC1L1*	5′–GCTGGCTGGCTCTCATCATCATC–3′	5′–AGCCTGCTGTCTTGTTCTTGTTCC–3′
*CYP7A1*	5′–CCCTCCAGGGAGATGCTCTGTG–3′	5′–ACATCCCTTCCGTGACCCAGAC–3′

#### Protein Preparation and Western Blot Analysis

These procedures were performed as previously described (Bolsoni-Lopes et al., 2013). Tissues were dissociated using cell lysis buffer containing protease inhibitors (Qiagen, Hilden, Germany). The protein concentrations were measured using the BCA (Thermo Fisher Scientific, USA) method. The tissue protein extracts were separated on SDS-PAGE gels and electro-transferred onto polyvinylidene fluoride membranes (Bio-Rad, USA). Specific proteins were detected using the following specific antibodies: beta-actin (1:2,000, CST, 3700S), HMGCR (1:1,000, Abcam, ab174830), NPC1L1 (1:1,000, Abcam, ab121000), LXRα (1:1,000, Abcam, Ab176323), CYP7A1 (Abcam, ab65596). Secondary horseradish peroxidase-conjugated antibodies were used at 1:2,000 dilutions. The proteins were detected using Western Chemiluminescent HRP Substrate (Cell Signaling Technology, USA). All experiments were repeated at least three times.

#### Statistical Analysis

All the experimental data are presented as means ± standard deviations. Statistical significance was analyzed using one-way ANOVA and Tukey’s *post-hoc* test, as appropriate, in SPSS 23.0 (IBM, Inc., Armonk, NY, USA). *p* < 0.05 was considered to represent statistical significance.

## Results

### cis-palmitoleic acid, trans-palmitoleic acid and Mouse Body Weight

Body weight was measured once a week, and calculated the fat index at the end of the research. Body weight in the HFD group increased steadily over the study period (Table 2). Compared with the control group, the weight of the mice in the simvastatin group increased over the 4-week period while that in the *c*POA and *t*POA groups showed a decreasing trend, but there was no statistical difference. Fat index in *c*POA group showed an increasing trend, while that in the *t*POA group showed a decreasing trend.

**TABLE 2:** Body weights and fat index in each group.

Groups		Body weight (g)				Fat index (%)
		1 week	2 week	3 week	4 week	
Control		28.5 ± 1.77	28.12 ± 2.09	28.08 ± 1.99	30.95 ± 2.39	0.30 ± 0.11
HFD		67.36 ± 4.24**	67.54 ± 2.95**	68.56 ± 2.91**	70.82 ± 3.86**	3.76 ± 1.16**
Simvastatin		66.7 ± 7.46	67.52 ± 5.34	68.70 ± 6.39	68.70 ± 6.01	2.63 ± 1.37#
HFD + *c*POA	150 mg/kg	67.41 ± 5.95	66.32 ± 5.53	66.72 ± 7.10	69.35 ± 7.23	4.11 ± 1.25
	300 mg/kg	68.33 ± 3.24	67.54 ± 4.19	66.18 ± 5.23	67.38 ± 6.50	4.07 ± 0.91
HFD + *t*POA	150 mg/kg	67.43 ± 5.14	66.15 ± 5.06	67.44 ± 5.13	68.87 ± 6.51	3.31 ± 1.07
	300 mg/kg	66.69 ± 4.37	65.03 ± 4.39	66.01 ± 5.02	67.58 ± 5.69	3.30 ± 1.15

n = 7, **p < 0.01 vs. the control group, #p < 0.05 vs. the HFD group.

### Administration of trans-palmitoleic acid Suppresses Mouse Appetite

The weight of food intake (g/10g/d) of the mice in each group was measured over the 4-week period, the results of which are shown in Table 3. Food intake in the HFD group was significantly lower than that in the control group (*p* < 0.01). Food intake in the simvastatin group was significantly lower (*p* < 0.01) than that in the HFD group, which suggests that simvastatin reduces appetite. *t*POA administration also significantly reduced food intake (*p* < 0.01), and *c*POA at 300 mg/kg/mouse tended to reduce food intake (*p* = 0.07).

**TABLE 3:** Food intake for each group over the 4-week study period.

Groups		Food intake (g/10g/d)
Control		0.94 ± 0.012
HFD		0.73 ± 0.0080**
Simvastatin		0.64 ± 0.0026##
HFD *+ c*POA	150 mg/kg	0.66 ± 0.078
	300 mg/kg	0.59 ± 0.061
HFD *+ t*POA	150 mg/kg	0.64 ± 0.0075##
	300 mg/kg	0.59 ± 0.023##

n = 7, **p < 0.01 vs. the control group, ##p < 0.01 and #p < 0.05 vs. the HFD group.

### Administration of trans-palmitoleic acid Reduces Serum and Liver Cholesterol Concentrations

**FIGURE 1:**
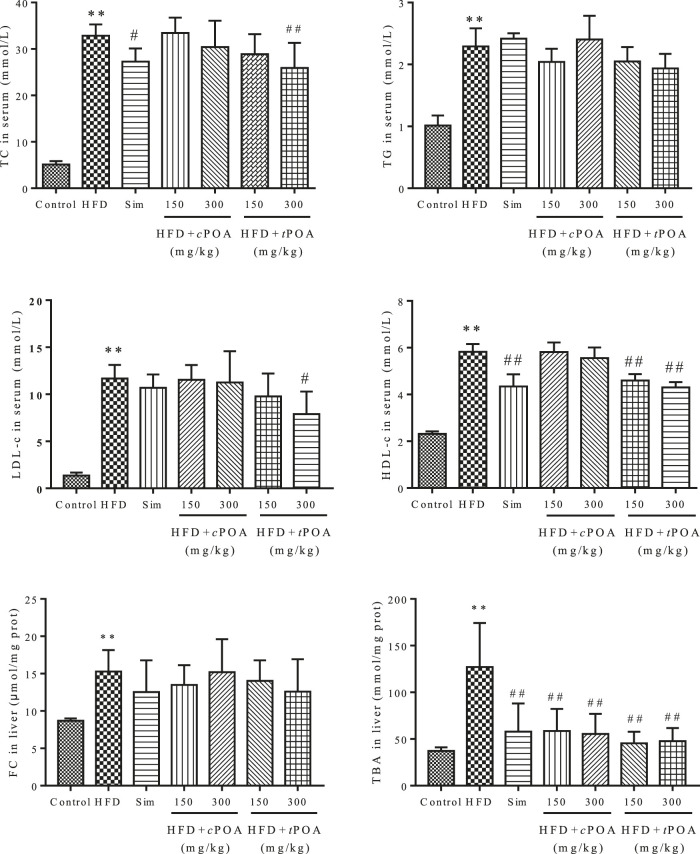
Effects of *c*POA and *t*POA on serum and liver lipid concentrations. *t*POA reduced the serum TC, HDL-c, and LDL-c concentrations and the liver FC and TBA concentrations. *c*POA reduced the TBA concentration in the liver (n = 7, **p < 0.01 *vs.* the control group, ##*p* < 0.01 and #*p* < 0.05 *vs.* the HFD group).

The TC, TG, LDL-c, HDL-c concentrations in the serum and FC and TBA concentrations in the liver increased more obviously (640.16, 226.73, 858.82, 251.29, 175.32, and 341.25%) in the HFD group than in the control group. Simvastatin clearly reduced the serum TC concentration (15.96%, *p* < 0.05) and HDL-c concentration (25.56%, *p* < 0.01). *t*POA significantly reduced the serum TC (21.71%, *p* < 0.05), LDL-c (32.53%, *p* < 0.05) and HDL-c (26.07%, *p* < 0.01) concentrations and reduced the TBA concentration (64.23%, *p* < 0.01) in the liver. However, *c*POA reduced the serum TC (7.43%, *p* = 0.99), LDL-c (3.68%, *p* = 0.98) and HDL-c (4.80%, *p* = 0.98) concentrations and liver FC (11.79%, *p* = 0.99) concentration, but there was no statistical effect. *c*POA significantly reduced the liver TBA concentration (56.39%, *p* < 0.01). (Figure 1).

### Administration of cis-palmitoleic acid Ameliorates Hepatic Steatosis

**FIGURE 2:**
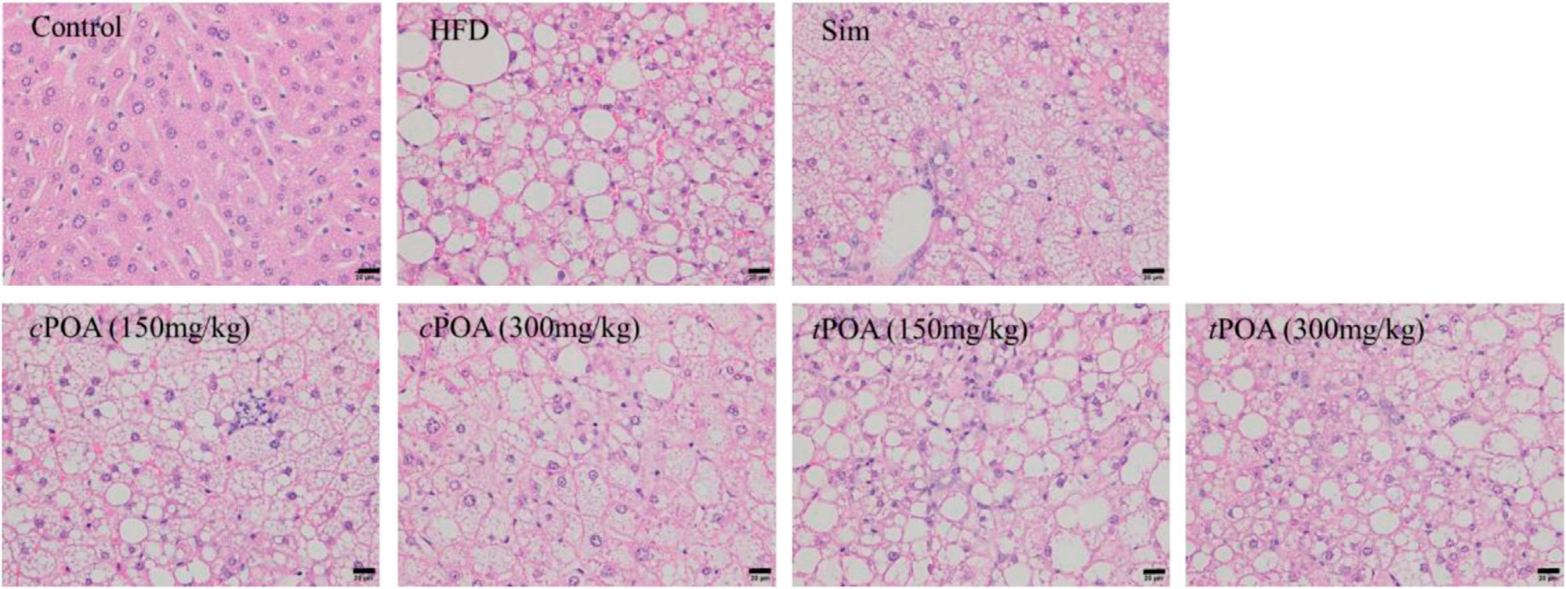
Effects of *c*POA and *t*POA on liver histology. Sections were stained with hematoxylin and eosin and examined at ×400 magnification.

Histological examination showed that mice in the control group each had a liver with a clear lobular structure that was irregularly arranged and radiated hepatocyte cords, with no obvious lipid deposition or congestion (Figure 2 and Table 4). In the HFD group, the boundaries of the hepatic lobules were unclear, the hepatocyte cords were disordered, the hepatic sinuses were narrow, and many hepatocytes were enlarged and contained lipid droplets and pale-staining cytoplasm. There was also obvious hepatic sinus congestion and inflammatory cell infiltration. In the simvastatin group, the arrangement of liver cell cords was more normal, the boundaries of the liver lobules were clearer, and there was less inflammatory cell infiltration, but intracellular lipid droplets were still abundant. The structure of the hepatic lobules in the *c*POA (150 mg/kg) group was better than in the HFD group, and in the *c*POA (300 mg/kg) group the hepatic lobular structure and arrangement of hepatocyte cords were significantly better, there were fewer intracellular lipid droplets, and there was less inflammatory cell infiltration and hepatic sinus congestion then seen in the control group. The histology of the *t*POA (150 mg/kg) group was very similar to that of the control group, while that of the *t*POA (300 mg/kg) group was slightly better, but there were still numerous intracellular lipid droplets and substantial inflammatory cell infiltration. Thus, *c*POA administration improved all aspects of the histology of the liver, whereas *t*POA administration improved the liver lobular structure, but did not significantly ameliorate the lipid deposition or the inflammation characterizing the HFD group.

**TABLE 4:** Liver pathology score.

Groups		Semi-quantitative data
Control		0.00 ± 0.00
HFD		3.00 ± 0.00**
Simvastatin		2.33 ± 0.50##
*c*POA	150 mg/kg	2.44 ± 0.53#
	300 mg/kg	1.78 ± 0.67##
*t*POA	150 mg/kg	3.00 ± 0.00
	300 mg/kg	2.67 ± 0.50

##p < 0.01 vs. the control group, **p < 0.01 and *p < 0.05 vs. the HFD group, n = 3.

Liver injury scores were assigned according to the cellular changes apparent in three randomly selected fields on each section (at ×400 magnification). The severity of the pathology was graded 0–3, with control liver cells being awarded a 0 grade. When a small number of hepatocytes were enlarged and each cytoplasm contained droplets, a grade of 1 was recorded. When most of the hepatocytes were enlarged and contained droplets, a grade of 2 was recorded. Finally, when there was extensive hepatocellular enlargement, with pale cytoplasm and numerous droplets, a grade of 3 was recorded. The scores for each group of mice are shown in Table 4. The result showed that *c*POA improved the structural features of liver tissue (40.67%, *p* < 0.01), as did *t*POA (11.00%), but with no statistical effect.

### 3-Hydroxy-3-Methyl Glutaryl Coenzyme Reductase Liver Expression Is Reduced in the cis-palmitoleic acid and trans-palmitoleic acid Groups

**FIGURE 3:**
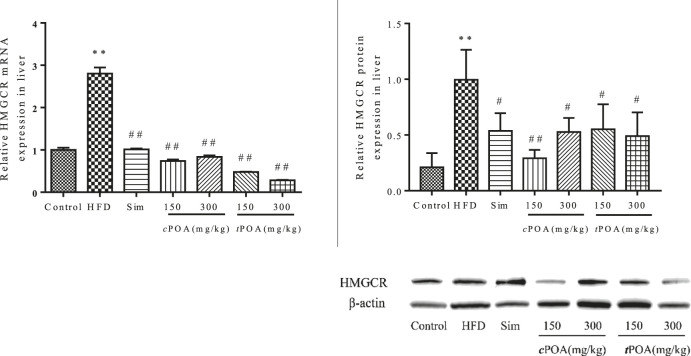
HMGCR gene expression and protein levels in each group (**, *p* < 0.01, *vs.* control, ##, *p* < 0.01, *vs.* HFD, n = 3).

The gene and protein levels of HMGCR in each group were evaluated by real-time PCR and western blotting (Figure 3). The results showed that the HMGCR gene’s transcript levels were significantly down-regulated in the *c*POA (73.02, 70.19%, *p* < 0.01) and *t*POA (82.94, 89.95%, *p* < 0.01) groups and that protein expression levels were reduced in the *c*POA (70.64, 47.11%, *p* < 0.01) and *t*POA (44.52, 50.65%, *p* < 0.01) groups, which indicates that cholesterol synthesis was inhibited in both groups.

### Intestinal Niemann-Pick C1-Like 1 Expression Is Reduced in the trans-palmitoleic acid Group

**FIGURE 4:**
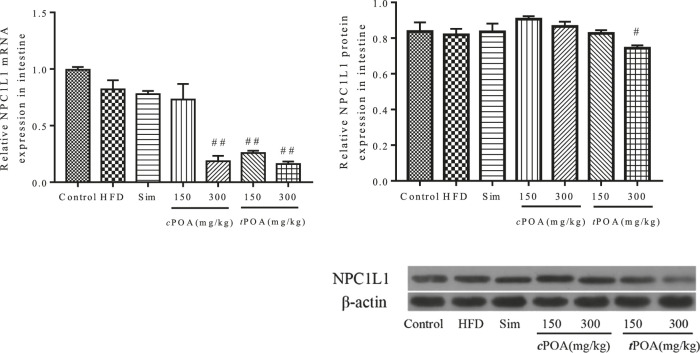
NPC1L1 gene and protein levels in each group (**, *p* < 0.01, *vs.* control, ##, *p* < 0.01, *vs.* HFD, n = 3).

Real-time PCR and western blotting analyses showed that the intestinal expression of NPC1L1 protein in the HFD group did not statistically differ from that of the control group (Figure 4). The NPC1L1-specific intestinal mRNA and protein expression levels in the simvastatin group did not statistically differ from those of the HFD group because this drug reduces serum cholesterol by inhibiting cholesterol synthesis rather than by inhibiting intestinal absorption or increasing cholesterol excretion (Shefer et al., 1970).

*t*POA administration significantly reduced the expression of NPC1L1 protein in the small intestine (9.16%, *p* < 0.01), which implies that it inhibited the absorption of cholesterol from the gut into the intestinal mucosa. The protein expression of NPC1L1 in the small intestine of the *c*POA-treated mice had no effect on NPC1L1 expression in the small intestine.

### Cholesterol 7-Alpha Hydroxylase -specific Liver Expression Levels Increased in the cis-palmitoleic acid and trans-palmitoleic acid Groups

**FIGURE 5:**
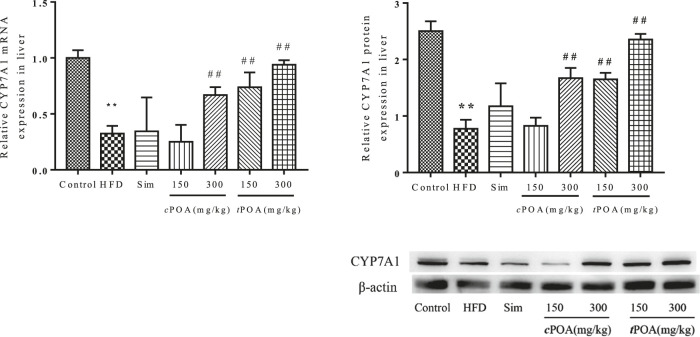
CYP7A1 gene and protein levels in each group (**, *p* < 0.01, *vs.* control, ##, *p* < 0.01, *vs.* HFD, n = 3).

CYP7A1 is a rate-limiting enzyme in the metabolism of cholesterol into BAs in the liver. CYP7A1 liver-specific protein expression in the HFD group was lower than that in the control group (Figure 5), which suggests that cholesterol metabolism was lower in this group and that cholesterol would likely accumulate in the liver. CYP7A1 expression in the simvastatin group was higher (1.94%) than in the HFD group. The 300 mg/kg *c*POA group and the two *t*POA groups (150 mg/kg and 300 mg/kg) showed upregulation of CYP7A1 (37.12, 41.33, 61.52%, respectively), but the 150 mg/kg *c*POA group showed no statistical difference in CYP7A1 expression, which suggests that a high dose of *c*POA or *t*POA can reduce the cholesterol concentration by upregulating CYP7A1 expression to increase its excretion in the form of BAs.

### LXRα-specific Liver Expression Increased in the trans-palmitoleic acid Group

**FIGURE 6:**
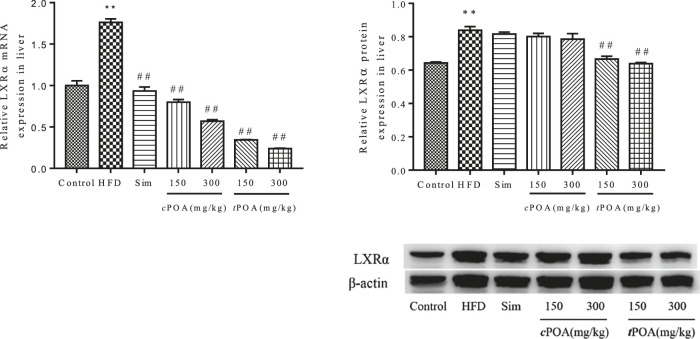
LXRα gene and protein levels in each group. (**, *p* < 0.01, *vs.* control, ##, *p* < 0.01, *vs.* HFD, n = 3).

The hepatic gene and protein expression of LXRα in the HFD group was significantly higher (*p* < 0.01) than that in the control group (Figure 6). *c*POA at 150 and 300 mg/kg and *t*POA at 150 and 300 mg/kg decreased LXRα mRNA production by 54.61 and 67.69% and by 80.48 and 86.39%, respectively. Simvastatin had no statistical effect on LXRα expression. *t*POA at 150 and 300 mg/kg dose-dependently reduced the expression of LXRα (20.65, 23.93%, respectively, *p* < 0.01), but *c*POA (2.74, 4.55%, respectively) had no statistical effect.

## Discussion

**FIGURE 7:**
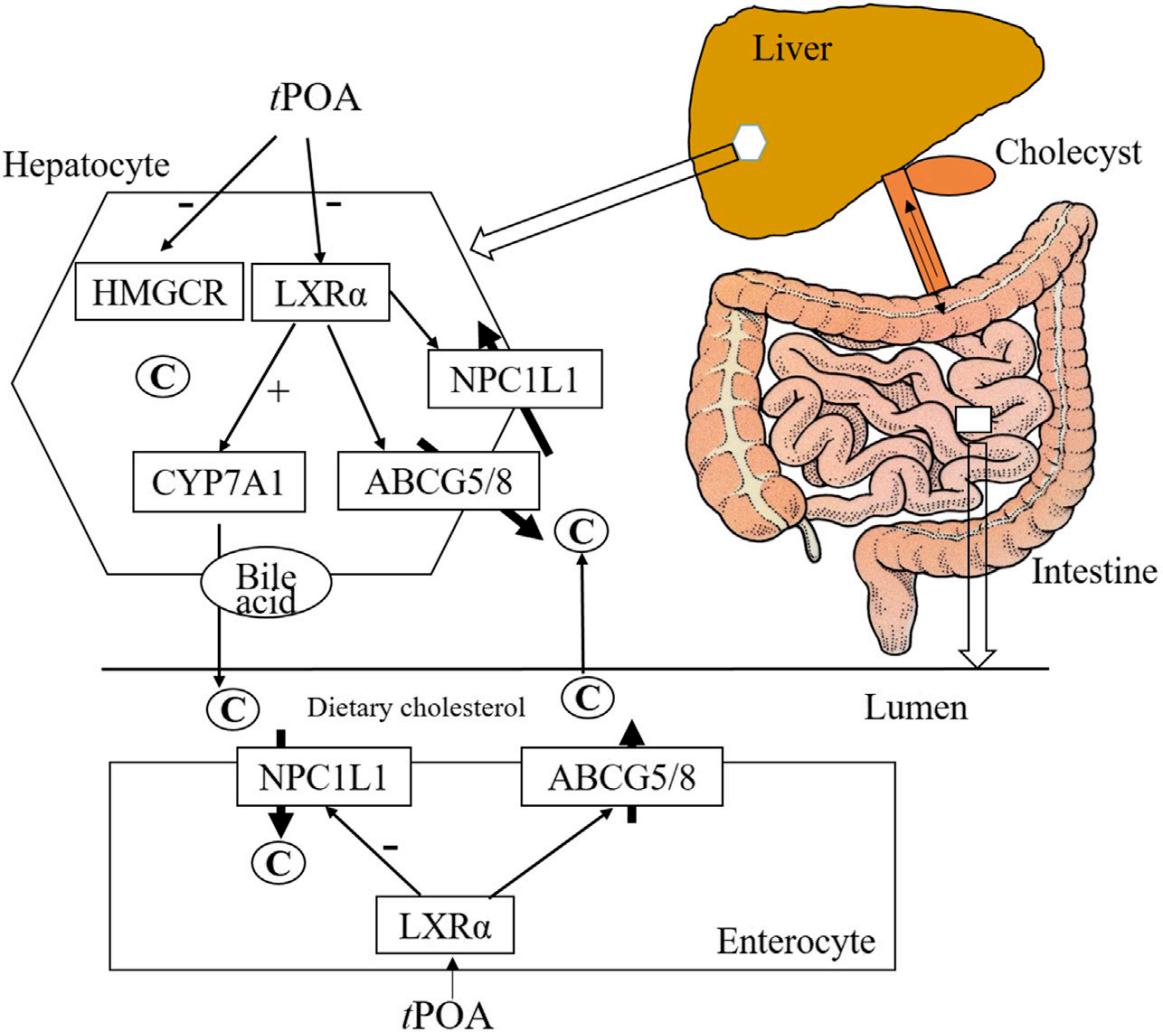
Hepatic and intestinal gene and protein expression. *t*POA administration reduced LXRα and NPC1L1 mRNA expression and protein levels, and increased CYP7A1 liver-specific mRNA and protein expression (n = 3). *c*POA had no effect on LXRα protein expression, but reduced NPC1L1 mRNA and protein expression and increased CYP7A1 mRNA and protein expression when administered at 300 mg/kg n = 3. ##*p* < 0.01 *vs.* the control group; **p < 0.01 and *p < 0.05 *vs.* the HFD group.

POA is a monounsaturated fatty acid found in fish oil, hippophae fruit oil, and olive oil. In this study, the effects of *c*POA and *t*POA isomers on the metabolism and intestinal absorption of cholesterol were compared in hypercholesterolemic mice, as summarized in Figure 7. *t*POA administration significantly reduced food intake, a finding consistent with the results of a previous study (Myant and Mitropoulos, 1977), while *c*POA had no significant effect. Our data show that *t*POA significantly reduced the serum TC and HDL-c concentrations and reduced the hepatic concentrations of FC and TBA. This indicates that *t*POA is more effective than *c*POA at ameliorating hypercholesterolemia and that this amelioration is likely achieved by regulating cholesterol metabolism via cholesterol synthesis reduction and absorption and by increasing cholesterol excretion, LXRα as their possible target.

The body weight in *c*POA group had no significant change, and reduced the hepatic steatosis, but fat index of that showed an increasing trend; The obvious interpretation was that the hepatic fat was mobilized by *c*POA and stored in the adipose tissues. It was especially relevant given that some reports had shown a positive correlation between serum concentration of *c*POA and adiposity, high level TG (Djousse et al., 2012; Luan et al., 2018; Trico et al., 2020). While, the body weight and fat index in *t*POA group showed decreasing trend, but had no significant effect on hepatic steatosis, therefore we speculated *t*POA increased hepatic lipid oxidation and export and increased thermogenesis in the brown adipose tissues (Wang, 2008).

Cholesterol in serum comes from (endogenous) synthesis and (exogenous) absorption. Most cholesterol (70–80%) is synthesized in the liver, Acetyl CoA is the direct raw material of cholesterol synthesis, which comes from glucose, fatty acid and some amino acid metabolites (Grundy et al., 1969; Li and Jiang, 2011). HMGCR is a rate-limiting enzyme in cholesterol synthesis in this organ. Cholesterol synthesis is activated by consuming high-fat food, which is the primary source of cholesterol from outside of the body (David and Spady, 1992; Feng and Xiao, 2010). *c*POA and *t*POA were both found to reduce HMGCR gene transcription and protein expression levels and cholesterol synthesis decreased in the liver; therefore, the FC concentration in the liver and TC in the serum were reduced also.

The efficiency of cholesterol absorption depends on the inflow and outflow of cholesterol molecules across the brush border membrane of enterocytes (Wang et al., 2017). NPC1L1 mediates the absorption of cholesterol from dietary components and BAs into intestinal cells and is therefore involved in the regulation of cholesterol and fatty acid metabolism and the enterohepatic circulation. NPC1L1 overexpression increases small intestinal epithelial cells’ absorption of cholesterol from the diet and BAs (Wang and Tang, 2009). Furthermore, several studies have shown that cholesterol absorption is reduced by 90% when NPC1L1 is knocked out in mice (Davis et al., 2004). In the present study, *t*POA administration reduced NPC1L1 protein expression in the intestine, alongside a reduction in food intake. Conversely, *c*POA administration had no effect on protein NPC1L1 levels in the intestine, which suggests that *t*POA may reduce the absorption of cholesterol in this organ, but *c*POA did not affect intestinal cholesterol absorption via NPC1L1.

Conversion of cholesterol to bile salts provides the major route for cholesterol elimination from the body. The initial and rate-limiting step in this pathway is catalyzed by 7ahydroxylase (Luan et al., 2018; Trico et al., 2020). The liver converts cholesterol into BAs via a series of reactions, including esterification. BAs are stored or excreted via the gut, where they are involved in intestinal lipid absorption. However, in insulin resistant states, the hepatic synthesis and secretion of BAs are both lower (Voss et al., 2016), which accords with CYP7A1 protein expression in the HFD group being lower than in the control group. *t*POA administration (150 and 300 mg/kg) or *c*POA administration (300 mg/kg) increased CYP7A1 mRNA and protein expression, which was expected to increase the hepatic metabolism of cholesterol to form BAs and reduce cholesterol accumulation in the liver.

About 95% of the bile acid absorbed in the intestinal from bile duct secretion, mainly in the form of combined BA in terminal ileum by apical sodium-dependent BA transporter (ASBT), via the portal circulation to the liver and secreted by the liver, called the process of the enterohepatic circulation of BAs (Djousse et al., 2012). CYP7A1 protein expression in HFD group decreased, but TBA in liver raise significantly, it probably that the BA synthesized in the liver couldn’t excrete in time or increase the BA circulation to the liver. TBA in Sim, *c*POA and *t*POA group were reduced significantly, it possible that they increased the excretion of BAs in liver, reduce the absorption in intestine, and regulated metabolism balance the enterohepatic circulation (Figure 7).

We have previously demonstrated the coordinated regulation of NPC1L1, a sterol influx transporter, and ABCG5/G8, sterol efflux transporters (Li et al., 2019). The latter two molecules form a heterodimer that promotes the metabolism of liver sterols into BAs and inhibits the absorption of sterols in the small intestine, thereby promoting cholesterol excretion (Ge et al., 2008; Hong and Tontonoz, 2014; Wang et al., 2015; Huang et al., 2018c). ABCG5 and ABCG8 are members of the ATP-binding cassette subfamily of transmembrane transporters and are mainly expressed in intestinal epithelial cells and liver cells. Therefore, the reduced TBA concentrations seen in the liver in the *c*POA and *t*POA groups are related to CYP7A1 and ABCG5/G8.

LXRs are involved in the hepatic synthesis of BAs and in cholesterol homeostasis, and PPARs play a central role in cholesterol absorption, outflow, and excretion (Jehad et al., 2019; Nakajima et al., 2019). In rodents, CYP7A1 is an LXRα target gene; therefore, when LXRα is knocked out in mice, the consumption of a HFD does not upregulate the expression of CYP7A1, but the circulating cholesterol concentration increases significantly (Li et al., 2019). Additionally, as previously reported, NPC1L1 deficiency in C57BL/6 mice reduces hepatic lipid accumulation and other pathological changes induced by LXR agonists (Tang et al., 2008). Furthermore, NPC1L1 expression is induced by an LXRα receptor agonist in Caco2 cells (Li et al., 2014). Another study showed that activated LXRα protein regulates both NPC1L1 and ABCG5/G8 expression to promote cholesterol metabolism (Li et al., 2018). Freeman *et al.* (Freeman et al., 2004) also found that LXR induces ABCG5 and ABCG8 expression, which regulates cholesterol absorption in the small intestine. In the present study, *t*POA inhibited the expression of LXRα, whereas *c*POA had no effect on LXRα expression. This implies that *t*POA affects LXRα transcription and therefore regulates the expression of downstream enzyme and lipid metabolism genes.

In summary, *t*POA regulated the expression of HMGCR, NPC1L1and CYP7A1 in the mouse liver and intestine, and inhibited the expression of LXRα as the target, which we would expect to reduce the synthesis in liver, intestinal absorption of cholesterol and increase biliary excretion, thereby reducing the hepatic and serum cholesterol concentrations. Conversely, we found evidence that doses of 150 or 300 mg/kg *c*POA reduced cholesterol accumulation in the liver by reducing food intake and increasing BA synthesis in the liver, while having no effect on intestinal absorption. In addition, *c*POA implied a pro-adipogenic effect, while that *t*POA had a trend of reducing adipose tissues. Further studies are required to thoroughly elucidate the mechanisms of the anti-hypercholesterolemic effects of *c*POA and *t*POA.

## Data Availability Statement

The raw data supporting the conclusions of this article will be made available by the authors, without undue reservation, to any qualified researcher.

## Ethics Statement

All animal experiments were performed in accordance with the guidelines of Nanjing University of Traditional Chinese Medicine for the ethical care of animals and approved by the Medical Ethics Committee of Nanjing University of Traditional Chinese Medicine (No. 201811A027). The study was carried out in accordance with the National Institutes of Health Guide for the Care and Use of Laboratory Animals (NIH Publications No. 8023, revised 1978).

## Author Contributions

WH conceptualized the study, contributed to the methodology and experiments, wrote and reviewed the original draft, and acquired the funding. BH, KB contributed to the experimental work, and wrote, reviewed and edited the manuscript. RT contributed to the methodology and experimental work, and wrote, reviewed and edited the manuscript. TY contributed to the methodology and experimental work, curated and analyzed the data, reviewed and edited the manuscript, and acquired the funding. JS curated and analyzed the data, contributed resources, and wrote, reviewed and edited the manuscript. RY analyzed the data, contributed resources, and wrote, reviewed and edited the manuscript. HW conceptualized the study, contributed to the experimental work, wrote and reviewed the original draft, validated the data, supervised and administered the project, and provided resources.

## Funding

This work was supported by the Scientific Research Foundation of the Third Institute of Oceanography, State Oceanic Administration, China (grant number 2016040), Agricultural guidance projects of Fujian Province, China (grant number 2017N0017), Science and Technology Plan Program of Fujian Province, China (grant number 2017Y9056), the National Natural Science Fund, China (grant number 81870138).

## Conflicts of Interest

The authors declare that the research was conducted in the absence of any commercial or financial relationships that could be construed as a potential conflict of interest.
